# Cardiac cycle weighted ΜQFR for the assessment of functional relevance of the myocardial bridge

**DOI:** 10.1007/s10554-025-03420-y

**Published:** 2025-05-27

**Authors:** Áron Üveges, András Ágoston, Balázs Tar, Boglárka Tamás, Mátyás Magyari, Stanisław Bartuś, Shengxian Tu, Zsolt Kőszegi

**Affiliations:** 1 Division of Cardiology, Department of Cardiology, Faculty of Medicine, University of Debrecen, Debrecen, Hungary; 2 Department of Cardiology, Szabolcs-Szatmár-Bereg Country Hospitals and University Teaching Hospital, Nyíregyháza, Hungary; 3 Kálmán Laki Doctoral School of Biomedical and Clinical Sciences, University of Debrecen, Debrecen, Hungary; 4 Institute of Cardiology, Jagiellonian University Medical College, Kraków, Poland; 5 Department of Cardiology and Cardiovascular Interventions, University Hospital, Kraków, Poland; 6 Biomedical Instrument Institute, School of Biomedical Engineering, Shanghai Jiao Tong University, Shanghai, China

**Keywords:** Angina pectoris, Angiography, Coronary function testing, Fractional flow reserve, Myocardial bridging, Quantitative flow ratio, Hydrostatic pressure

## Abstract

Myocardial bridging (MB) causes coronary artery compression in systole, potentially resulting in angina and arrhythmias. Stent implantation in patients with MB may offer short-term improvement of symptoms, but carries a high risk of restenosis. We present a symptomatic patient with MB who was assessed by a novel angiography-based functional index that incorporates both the diastolic and systolic phases of the heart cycle – termed “cardiac cycle weighted µQFR” (µQFRccw). In addition to the observed dynamic systolic narrowing of the left anterior descending artery (LAD), a fixed stenotic (atherosclerotic) component was suspected due to mild diastolic diameter reduction. Invasive physiological measurements during adenosine and dobutamine provocation were also performed. Following administration of 200 µg ic. adenosine, the FFR was measured as 0.74, while during a 40 µg/bwkg/min dobutamine iv. infusion it was measured as 0.83. The measured FFR during adenosine-induced hyperemia was found to be very close in value to the calculated µQFRccw (0.74 vs. 0.75). This case demonstrates the potential utility of the image-based cardiac-cycle weighted µQFR in MB evaluation. In our opinion, the discordance between the significant invasive adenosine FFR and the non-significant dobutamine FFR results may indicate a fixed atherosclerotic component of the lesion, which could support the consideration of stenting despite the potential risk of restenosis.

## Learning points

•In myocardial bridging atherosclerosis generally spares the compressed segment, but in some cases, it may coexist and contribute to obstruction of epicardial flow.

•This case demonstrates the feasibility of the image-based cardiac-cycle weighted functional assessment of the myocardial bridging using µQFR calculations during both diastolic and systolic frames.

•In the case of a myocardial bridge, a comparison of dobutamine and adenosine provocation may shed light on the fixed and dynamic components of the lesion.

## Introduction

Myocardial bridging (MB) is a common congenital anomaly characterized by a segment of a coronary artery that courses intramurally through the underlying myocardium. During systole, the myocardium may compress the affected coronary segment, potentially leading to angina, arrhythmias, or even sudden cardiac death [1]. While atherosclerosis typically spares the tunnelled segment, it can occasionally develop. However, distinguishing between symptoms arising from the dynamic component of MB and those due to coexisting atherosclerosis remains challenging, even with intracoronary physiological measurements.

Pharmacotherapy of symptomatic MB consists of beta-blockers and calcium channel blockers. Coronary artery bypass graft surgery and surgical myotomy may be considered in exceptional cases with severe symptoms despite medical therapy [2, 3]. There are currently no well-defined protocols for the invasive and surgical treatment of MB. In severely symptomatic patients, stent implantation can immediately improve the epicardial flow and provide symptom relief. However, there is a risk of perforation during stent deployment and an increased frequency of restenosis in the long term [4].

## Case summary

We present the case of a 51-year-old male patient with new-onset atrial fibrillation following Covid-19 virus infection. Echocardiography showed good left ventricular function, a slightly enlarged right ventricle, and slightly elevated pulmonary pressure. Stress ECG did not suggest significant myocardial ischemia. However, coronary angiography was indicated due to his symptoms of weakness and shortness of breath.

The angiography was recorded at 15 frames/s and showed a stenosis complicated with a bridge phenomenon in the medial section of LAD, which caused a 60% diameter reduction in systole and 40% in diastole according to visual assessment.

The calculation of µQFR (quantitative flow reserve with Murray bifurcation fractal law algorithm) was based on the diastolic and systolic frames of the invasive coronary angiography performed by the AngioPlus Core, V2 software (Shanghai Pulse Medical Technology Inc, Shanghai, China). The software automatically identifies and outlines the contours of the main coronary artery lumen and its branches. Consequently, based on the Murray bifurcation fractal law, a stepdown reference diameter function is generated.

The contrast flow velocity was computed by dividing the centerline length by the time of the contrast front spreading to the distal reference endpoint during the resting angiography, and simulated hyperemic flow was defined by an extrapolation derived from the previous database [5].

**Fig. 1:**
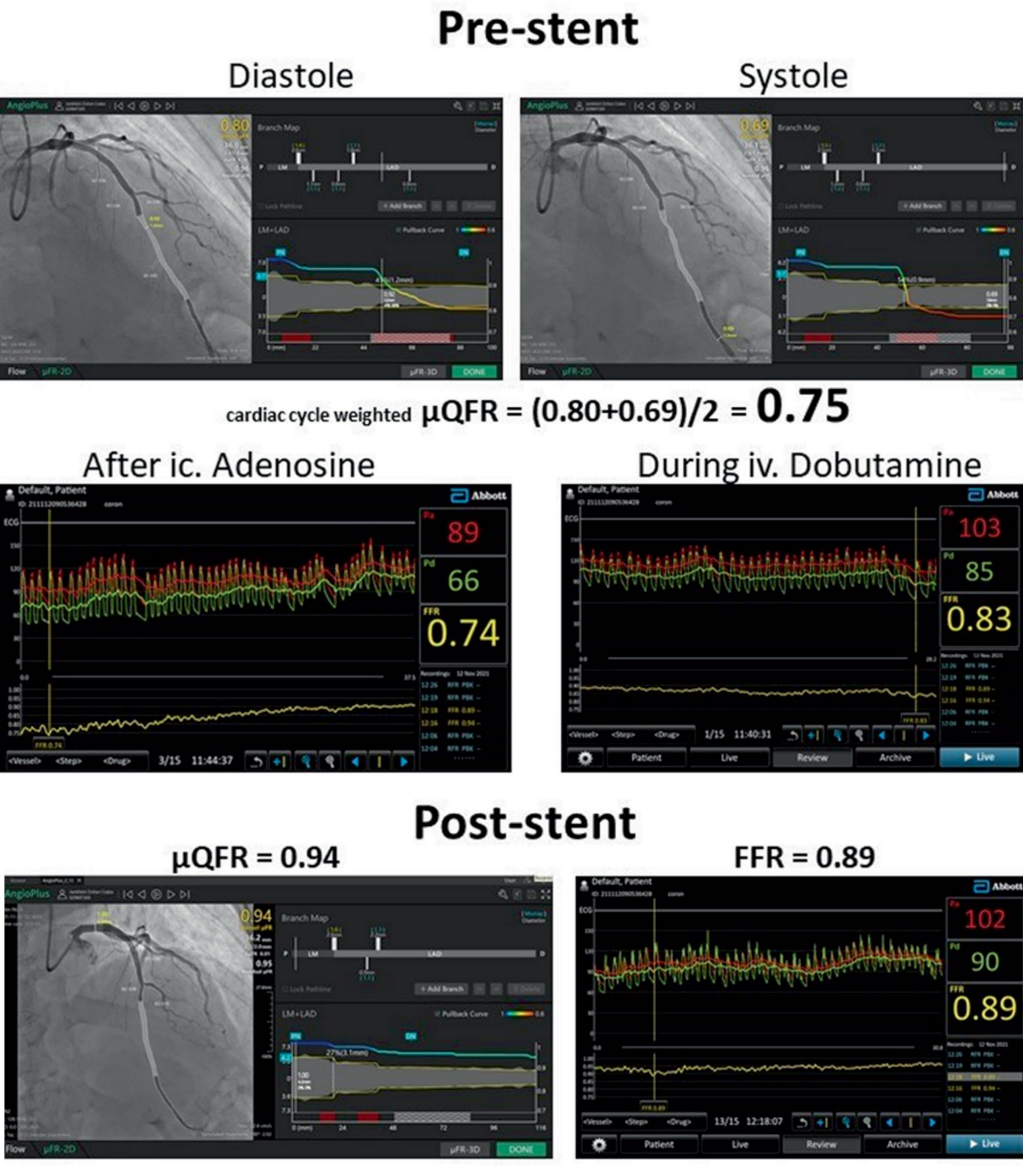
Pre-and post-stent µQFR calculations and invasive FFR measurements

In line with the previous recommendation [6, the average QFR value was obtained by equally weighing the diastolic and the systolic components of coronary flow, using a 1:1 ratio of diastolic µQFR to systolic µQFR. The measured “cardiac cycle weighted” µQFR (µQFRccw) proved to be significant (0.75) in our patient. (Fig. 1).

Physiological measurements after intracoronary adenosine provocation (200 µg ic.) resulted in an FFR of 0.74 and a resting Pd/Pa of 0.86. To assess the impact of the myocardial bridge, FFR was also measured under pharmacological stress using dobutamine (40 µg/kg/min) and atropine (1 mg), following the recommended protocol. This resulted in a non-significant FFR value of 0.83. The hemodynamic parameters measured during the resting state, after intracoronary adenosine injection and during dobutamine infusion are indicated in Table 1.

Based on these physiological results, sirolimus-eluting stent implantation (2.5/36 mm Supraflex Cruz, Sahajanand medical technologies ltd., India) was performed, and poststent measurements again showed similar ΜQFRccw and invasive FFR values (0.94 vs. 0.89) (Fig. 1).

**Table 1:** Hemodynamic parameters during the physiological measurements

	Heart rate (/min)	Systolic aortic pressure (mm Hg)	Diastolic aortic pressure (mmHg)	Average aortic pressure (mmHg)
Resting condition	96	125	90	103
After adenosine	82	119	82	89
During dobutamine	141	162	101	129

## Discussion

Myocardial ischemia occurs in MBs due to systolic compression and delayed diastolic vascular relaxation, but concomitant atherosclerosis can also play a role in the resulting myocardial ischemia. Intravascular ultrasound imaging could be useful for imaging the lesion characteristics and the dynamic lumen changes during the cardiac cycle, but determining the exact hemodynamic consequence of the stenosis requires direct flow measurements or, as a surrogate for flow, intracoronary pressure measurements. A reliable assessment model is desirable for clinical decision-making in patients with MB. Invasive FFR has been proposed to evaluate the functional significance of coronary flow obstruction in MB.

It was found that the use of dobutamine rather than adenosine as a stressor agent during FFR measurements increases the sensitivity for the detection of ischemia in MBs due to the positive inotropic effect of dobutamine promoting further vessel compression [7].

In our case, we found higher FFR during dobutamine than after the ic. adenosine bolus as a seemingly paradoxical behaviour of the coronary lesion at the site of the bridge segment. One possible factor to explain this paradox could be that following dobutamine infusion, the artificial reduction in systolic pressure gradients due to distal systolic pressure overshooting can even decrease the average pressure gradient [8]. On the other hand, it is also worth noting that we found higher Pa during the dobutamine infusion than after the ic. adenosine bolus, therefore similar pressure gradients were detected during the two measurements (Pa-Pd = 129 − 107 = 22 mmHg and 89 − 66 = 23 mmHg). In our opinion, this observation does not favour the dominant component of the flow limitation from the dynamic myocardial bridge during the increased contractility. Instead, we propose that the concomitant fixed atherosclerotic component may play a role in the ischemic FFR value.

As a previous Doppler study demonstrated MB “creates an area of low wall shear stress that has been posited to explain the development of atherosclerotic plaque” [9].

Although the less invasive Quantitative Flow Ratio (QFR) is not specifically designed to assess FFR in cases of myocardial bridging (MB), it may offer potential as an alternative evaluative tool. Given the dynamic nature of stenosis in MB, QFR calculation must be based on the specific geometric configuration of the vessel during the cardiac cycle. Previous studies have proposed the use of a time-averaged QFR, calculated as the arithmetic mean of systolic and diastolic QFR values. We adopted this method and referred to the resulting parameter as µQFRccw, which demonstrated a good agreement with invasive FFR measurements (Fig. 1).

Before the stent implantation, the µQFR was calculated as the average of the diastolic and the systolic values (“cardiac cycle weighted” µQFR: µQFRccw = 0.75). The invasive FFR after administration of 200 µg ic. Adenosine was 0.74, while an FFR of 0.83 was recorded during 40 µg/kg/min dobutamine (+ 1 mg atropine) iv. infusion.

After stent implantation, µQFR was calculated as 0.94 while the invasive FFR after ic. adenosine was 0.89.

## Conclusions

Our case demonstrates the feasibility of the image-based, cardiac-cycle weighted functional assessment in the evaluation of MB. The discordance between significant invasive adenosine FFR after adenosine administration and the non-significant FFR under dobutamine may suggest a fixed atherosclerotic component of the MB. This finding may support the consideration of stenting, despite the potential risk of restenosis.

## Data Availability

No datasets were generated or analysed during the current study.
